# Relationships between Different Field Test Performance Measures in Elite Goalball Players

**DOI:** 10.3390/sports7010006

**Published:** 2018-12-28

**Authors:** Gabriel Goulart-Siqueira, Stefano Benítez-Flores, Alexandre R. P. Ferreira, Alessandro M. Zagatto, Carl Foster, Daniel Boullosa

**Affiliations:** 1 Physical Education, Catholic University of Brasilia, Águas Claras 71966-700, Brazil; gabrielpolui@hotmail.com (G.G.-S.); stefanobenitez@gmail.com (S.B.-F.); alexandreispf@gmail.com (A.R.P.F.); 2 Laboratory of Physiology and Sport Performance (LAFIDE), Bauru 17033-360, Brazil; azagatto@yahoo.com.br; 3 Department of Exercise and Sport Science, University of Wisconsin-La Crosse, La Crosse, FL 54601, USA; cfoster@uwlax.edu

**Keywords:** visual impairment, physical fitness, aerobic capacity, anaerobic capacity, sports physiology

## Abstract

Goalball is a Paralympic sport involving people with visual impairment. Little is known about the physical fitness of elite players of this sport, as previous studies only evaluated body composition and aerobic capacity. Thus, the aim of this study was to describe the performance of elite goalball players in different physical tests and to look for relationships between them. Eleven elite Brazilian goalball players, seven males and four females, were evaluated for body composition, maximal handgrip isometric force (MHGF), countermovement jump (CMJ), throwing velocity (TV) and the Yo-Yo intermittent recovery test level 1 (Yo-Yo IR1). Players produced 41.54 ± 8.41 kgf in MHGF, 34.81 ± 7.2 cm in CMJ, 14.21 ± 1.89 m∙s^−1^ in TV, and 505 ± 313 m in Yo-Yo IR1, with an estimated maximum oxygen consumption (VO_2_max) of 40.64 ± 2.63 mL∙kg^−1^∙min^−1^. Most performance tests exhibited moderate to high correlations among them, while the percentage of body fat correlated with both the CMJ and Yo-Yo IR1 results. The current study reveals that CMJ could be a valuable monitoring tool as it was correlated with all other performance tests. The highest correlation observed was with TV (r = 0.754; *p* < 0.05), which is a key capacity in goalball. Moreover, high levels of body fat could be detrimental to anaerobic performance.

## 1. Introduction

Goalball is a Paralympic team sport with three visually impaired players in each team, which is played on a standard volleyball court. Irrespective of their visual impairment, all players wear blackened eye goggles for standardization. The objective is to throw the ball into the opponents’ goal [1]. The ball emits sound because it has bells embedded within it, allowing players to identify the location of the ball at every moment. This demands auditory, spatial and tactile awareness [2]. Due to these stimuli and the motor requirements, goalball provides an important vehicle for the immersion of people with visual impairments into sport [3].

Previous studies have been conducted to identify motor fitness and isokinetic strength [2,4,5], aerobic capacity [6], body composition [7,8,9], postural stability and static balance [10,11], throwing performance [12,13,14], and game performance [15] in goalball players of different ages, sexes and skill levels. However, there are few studies [6,7,8] describing the physical fitness components of elite adult players. For instance, Gulick and Malone [6] validated a field beep test for evaluating the aerobic capacity of Paralympic female athletes and showed a low aerobic capacity of these athletes. Subsequently, Romanov et al. [8] identified some correlations between the ratio of waist to hip circumference with the ranking of adult male teams. However, Aslan et al. [7], in a more recent study, did not confirm any difference between successful and unsuccessful male teams with respect to body composition.

Given this important lack of information on the physical fitness components of adult athletes, which does not allow comparisons between athletes of different sex and skill levels, or to identify the best evaluations for training monitoring, we conducted this descriptive study with a sample of elite male and female athletes. The first objective of this study was to describe the performance of athletes in different physical tests conducted in the field. Secondly, we aimed to look for correlations between the tests selected to identify the most efficient evaluations to monitor training. We hypothesized that most tests would be correlated amongst each other.

## 2. Materials and Methods

### 2.1. Participants

Eleven first level Goalball players, including seven males (age 25.5 ± 7.0 years) and four females (age 25.2 ± 5.4 years), were recruited from a Brazilian first level Goalball team. The players trained ≥4 times/wk for ≥90 min, had an average experience in the sport of 7.27 ± 3.88 years, and competed regularly in the Brazilian championship. Five players were part of the national team, with experience in the Paralympic Games and World Cup. Five players were classified as B1 (totally or almost totally blind), two players as B2 (visual acuity ranging from 1.50 to 2.60 and/or a visual field constricted to a diameter of less than 10 degrees), and four players as B3 (partial sight, with visual acuity from 2/60 to 6/60). The players were informed about the study conditions and provided written informed consent. All procedures were performed in accordance with the Declaration of Helsinki, and the local university ethics committee approved the protocol.

### 2.2. Study Design

The study was conducted during the pre-season after 3 months of training, and included 2 days of evaluation separated by 72–96 h, and after a minimum of 24 h of rest. On the first day, participants were familiarized with each test. On the second day, participants were evaluated for body composition, maximal handgrip isometric force (MHGF), countermovement jump (CMJ), throwing velocity (TV) and Yo-Yo intermittent recovery test level 1 (Yo-Yo IR1).

### 2.3. Body Composition

Body mass (kg) (2096PP, Toledo, São Paulo, Brazil) and stature (cm) (ES-2040, Sanny Medical, São Paulo, Brazil) were measured, and subsequently the body mass index (BMI) was calculated. Seven skinfolds were recorded with a skinfold calliper (Lange Skinfold Calliper, Beta Technology, Houston, TX, USA), and the percentage of body fat (BF) was determined with the formula of Wilmore and Behnke [16].

### 2.4. Vertical Jump

The CMJ was used to determine the muscular power capacity of the lower limbs. The CMJ height was recorded with an iPad (iPad Air 2 A1566, Apple Inc., Cupertino, CA, USA) placed in front of the athlete, using the validated app "My Jump" (Apple Inc., Cupertino, CA, USA). The app records the flight time and then calculates the jump height by applying a standard kinematic formula [17]. Players performed two attempts, with 1 min of rest in between. The best jump was used for analysis.

### 2.5. Maximal Handgrip Isometric Force

The MHGF was recorded utilizing a validated dynamometer (Sammons Preston Rolyan, Jamar, Bolingbrook, IL, USA). The players sat, with their shoulders slightly adducted and neutrally rotated, and with their elbow flexed to 90°. They were given two attempts per hand, separated by 1 min of rest, to apply the maximum possible force. The highest achieved value with each hand was recorded and the average of both was used for further analysis.

### 2.6. Throwing Velocity

The maximum TV of the ball was measured during a simulation of a “penalty throw” following official rules, in which an athlete threw the ball after a run-up of ~6 m, from one side of the court to the other, where the 9 meter-wide goal was defended by a single opponent. TV was measured using a sports radar (Stalker ATS Pro II, Applied Concepts, Tully, NY, USA) placed a meter and a half behind the goal at 1 m of height. All athletes were encouraged to achieve the highest possible velocity with a standardized front throwing technique. Two penalty throws were evaluated with one minute of recovery between. The best was used for further analysis.

### 2.7. Yo-Yo Intermittent Recovery Test Level 1

The Yo-Yo IR1 is a practical test widely used to assess the ability to repeat intermittent high-intensity efforts in athletes of different levels [18]. The Yo-Yo IR1 consists of 2 × 20 m shuttle runs at increasing speeds interspersed with a 10 s period of active recovery. For the application of this test to goalball athletes, two researchers were placed at the beginning and the end of the 20 m path to call the players, allowing them to be oriented in a straight line. The test was finished when participants failed two consecutive times to reach the dictated speed. The total distance covered was considered the test result. The maximum oxygen consumption (VO_2_max) was estimated using the formula of Bangsbo et al. [18].

### 2.8. Statistical Analysis

Data are presented as means ± SD. Normality was assessed with the Shapiro–Wilk test. Pearson product-moment correlation coefficient (r) was used to assess the relationships between selected variables. All statistics were performed using appropriate software (IBM SPSS Statistics for Windows^®^, v. 20.0, Armonk, NY, USA). Figures were created in an Excel^®^ spreadsheet. The alpha level was set at *p* < 0.05.

## 3. Results

### Physical Fitness

Anthropometrics and physical performance measurements of all participants are presented in Table 1. All athletes performed all the tests except one male (Athlete 8) and one female (Athlete 4) who could not perform the TV test because of time constraints during evaluations.

**Figure 1:**
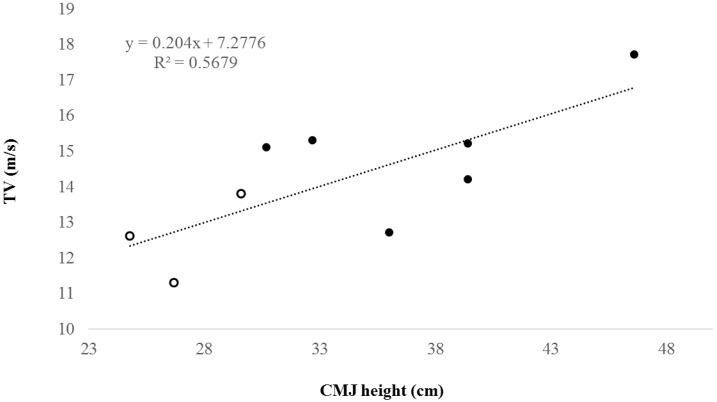
Relationship between maximum throwing velocity (TV) and countermovement jump (CMJ) height. Females are represented as white dots and males as black dots.

A matrix of correlations is presented in Table 2. Overall, most test results had moderate to high correlations, with R^2^ values lower than 0.4. The one exception was the highest correlation between TV and CMJ height, which resulted in a R^2^ of 0.57 (see Figure 1). In addition, some negative correlations were identified between BF and both CMJ and Yo-Yo IR1 performances.

## 4. Discussion

To the best of our knowledge, this is the first study evaluating different physical tests in the field in a sample of elite goalball athletes of both sexes. The results of these tests could serve as reference values for further evaluations, and for monitoring of goalball athletes in the field. Furthermore, the correlation among the results of most tests highlight the suitability of CMJ for monitoring goalball athletes, as performance in this simple test was consistently related to the other tests and, more importantly, was strongly related to TV, which is a key ability in goalball. In addition, some correlations were revealed between BF and both CMJ and Yo-Yo IR1 results, therefore suggesting the necessity of controlling BF for better outcomes.

Regarding body composition, the current results are in agreement with the previous literature [6,8]. Thus, it seems that being at the elite level is compatible with being overweight [6,8]. However, there is no consensus regarding the relationship between body composition parameters and performance level. Previously, Romanov et al. [8] identified some correlations between the ratio of waist to hip circumference with the ranking of adult male teams. In contrast, Aslan et al. [7] did not confirm any difference between successful and unsuccessful male teams with respect to body composition. In the current study, strong negative correlations were identified between BF and both the CMJ and Yo-Yo IR1 results. Despite the possible influence of sex on these results, it may be suggested that an excess of BF could have a negative influence on the anaerobic performance of these athletes. Given that goalball predominantly uses anaerobic alactic capacity [19], the reduction of BF could be necessary for elite goalball players, but not to the same extent as in other sports with greater locomotor demands that emphasise lactic and aerobic capacities. Therefore, future studies should verify if changes in body composition parameters, especially BF, could be related to better field performance at this elite level in both sexes, while identifying to what extent an excess of BF could be detrimental for goalball performance.

The CMJ results exhibited in the current study are better than those previously reported in a sample of sub-elite goalball players [2]. This may suggest that lower limb power is a desirable ability in this sport, which is characterized by brief explosive actions (i.e. throws and defensive actions) in which the energy contribution is provided by anaerobic alactic metabolism [19]. Further, CMJ performance was the physical ability most related to other tests (i.e. Yo-Yo IR1, TV and MHGF). This was in agreement with previous studies in other sports [20,21,22]. Moreover, a strong relationship between CMJ performance and TV was found (r = 0.754; R^2^ = 0.57), which is a key ability in goalball. This highlights the suitability of this simple and practical test for monitoring the training of goalball athletes on a daily basis. Future studies should verify the effectiveness of different training protocols for improving CMJ performance and, in turn, other fitness characteristics related to goalball performance in players of different levels.

As previously stated, TV is considered a key ability in goalball [14]. While previous studies have focused on time motion and technical aspects of throwing [12,14,15], to the best of our knowledge, this is the first study that has evaluated TV in a standardized format. While comparison with other sports is challenging and may be unnecessary due to a number of technical and tactical differences [23], further studies should verify the best physical and technical training protocols to improve this ability, while confirming the practicality of monitoring CMJ and other physical tests such as MHGF.

MHGF is considered an index of general muscle strength. This elite sample showed greater MHGF levels to those previously exhibited by a sub-elite sample [2]. This would signify that this test would likely be appropriate for evaluating goalball athletes of different levels. Meanwhile, performance in MHGF has exhibited the second highest correlation with TV (r = 0.667; *p* < 0.05), therefore suggesting a feasible alternative to CMJ evaluations. Future studies would help elaborate on the appropriateness of handgrip strength evaluations, while considering other handgrip performance parameters, such as the rate of force development (RFD), which could be more appropriate for neuromuscular evaluations of these athletes.

To the best of our knowledge, this is the first study evaluating Yo-Yo IR1 performance in goalball players. Thus, performance in this test is clearly below the levels of other elite athletes [18]. This may suggest that this test is not specific for goalball athletes. This could be explained by the fact that this test challenges both aerobic and anaerobic lactic metabolisms. Meanwhile, the estimated VO_2_max values calculated from this test are in agreement with the low values of the previous study by Gulick and Malone [6], which used an adapted beep test and suggested that aerobic capacity may not be very important for goalball performance. Further studies may elucidate the appropriateness of these and other tests for the evaluation of aerobic capacity in the field.

## 5. Conclusions

Our study revealed medium to high correlations between different field test results in a sample of elite goalball athletes. In addition, BF was also correlated with the CMJ and Yo-Yo IR1 results. Given the correlations of CMJ performance with other physical capacities, in particular with TV, the use of this simple test is suggested for the monitoring of goalball athletes. A reduction of BF in goalball athletes should also be recommended to improve anaerobic performance. Further studies should elaborate on the sensitivity of this and other tests to detecting performance changes in goalball athletes of both sexes and at different levels.

## Figures and Tables

**Table 1:** Physical characteristics and performance test results for all athletes.

Athlete	Sex	Age (yrs)	Height (m)	Body Mass (kg)	BMI (kg/m^2^)	BF (%)	MHGF (kgf)	CMJ (cm)	TV (m/s)	VO_2_max (mL/kg/min)	Yo-Yo IR1 (m)
1	F	22	1.54	62.6	23.27	26.4	35	29.6	13.8	41.1	560
2	F	33	1.53	59.4	25.37	34.5	27	26.7	11.3	37.4	120
3	F	21	1.67	89.3	32.01	37.7	37	24.8	12.6	38.1	200
4	F	25	1.66	57.5	20.86	16.6	34	31.7	-	40.4	480
5	M	21	1.76	77.2	24.92	18.2	42	36	12.7	42.1	680
6	M	36	1.68	74.4	26.36	25.2	37	32.7	15.3	40.1	440
7	M	20	1.8	84.3	26.01	23.4	53	39.4	14.2	39.4	360
8	M	21	1.65	61.3	22.51	5	43	45.4	-	47.1	1280
9	M	28	1.74	83.8	27.67	20.1	50	46.6	17.7	40.4	480
10	M	19	1.77	98.1	31.31	28.2	53	30.7	15.1	38.7	280
11	M	34	1.68	70.5	24.97	17	46	39.4	15.2	42.1	680
Mean SD		25.4	1.67	74.4	25.93	22.9	44	35.8	14.2	40.6	505
		6.2	0.09	13.5	3.40	9.1	9	7.2	1.9	2.6	313

F: female; M: male; BMI: body mass index; BF: body fatness; MHGF: maximal handgrip isometric force; CMJ: countermovement jump; TV: throwing velocity. VO_2_max: estimated maximum oxygen consumption. Yo-Yo IR1: total distance covered in the Yo-Yo intermittent recovery test level 1.

**Table 2:** Matrix of correlations between body composition and performance test results.

Physical Fitness	BMI	BF	MHGF	CMJ	TV	Yo-Yo IR1
BMI	-	0.655 *	0.355	−0.288	0.107	−0.557
BF	0.655 *	-	−0.306	−0.796 *	−0.566	−0.903 *
MHGF	0.355	−0.306	-	0.614 *	0.667 *	0.176
CMJ	−0.288	−0.796 *	0.614 *	-	0.754 *	0.667 *
TV	0.107	−0.566	0.667 *	0.754 *	-	0.377
Yo-Yo IR1	−0.557	−0.903 *	0.176	0.677 *	0.377	-

BMI: Body mass index; BF: body fatness; MHGF: Maximal handgrip isometric force; CMJ: Countermovement jump; TV: Throwing velocity; Yo-Yo IR1: Total distance covered in the Yo-Yo intermittent recovery test level 1. * *p* < 0.05.
